# Increased Early‐Mortality in Children With Solid Tumors During the COVID‐19 Pandemic in a Middle‐Income Country

**DOI:** 10.1002/cam4.70483

**Published:** 2024-12-23

**Authors:** Oscar Ramirez, Vivian Piedrahita, Santiago Bolivar, Karina Grillo, Adriana Linares, Carlos Pardo, Martha Piña, Amaranto Suarez, Carlos A. Portilla, Jesus Ardila, Viviana Lotero, Luz A. Urcuqui, Angela Trujillo, Patricia Montenegro, Luis E. Bravo, Paula Aristizabal, VIGICANCER Working Group, Gisella Barros, Edgar Cabrera‐Bernal, Angélica Castillo, Mauricio Chaparro, Marcela Estupiñán, Jessica Flechas, Jimmy Lagos, Jorge Hernández, John Lopera, Ingrid Aristizabal, Agustín Contreras, Iliana C. De los Reyes, Natalia González, Paula Guzman, Alejandra Calderón, Oscar González, Mauricio Mesa, Carolina Casas, Ana M. Infante, Leila Martínez, Angelica Castillo, Giovanny Rincón, Diego I. Estupinan‐Perico, María P. Obregón, David J. Garay, Julio A. Ropero, María A. Pérez, Liliana Barragán, Remberto Osuna, María X. Castro, Pamela A. Rodríguez, Diego Medina, Alexis Franco, Luis H. Romero, Ruth Castro, Carlos E. Narváez, Diana S. Rendón, Ángel Castro, Ayslin González, Heidy Marsiglia, Soraya Paternina, Francy H. Ortiz, Daniel Ozaeta, Bibiana Villa, Javier E. Fox, María R. Pérez, Carlos E. Restrepo, Lina M. Quiroz, Natalia Valencia, Alexandra Restrepo, Federico Arroyave, Hernán D. Vásquez, Gloria E. Suarez, Diana L. Valencia, Fabio J. Molina, Soraya Montaño, Aide Peña, José A. Cabezas, Nelson Ramírez, Maria del Rosario Álvarez

**Affiliations:** ^1^ Fundación POHEMA Unidad de Investigación Cali Colombia; ^2^ Clínica Imbanaco—Grupo Quirón Salud Unidad de Oncología y Hematología Pediátrica Cali Colombia; ^3^ Registro Poblacional de Cáncer de Cali, Departamento de Patología Universidad del Valle Cali Colombia; ^4^ Escuela de Enfermería Universidad del Valle Cali Colombia; ^5^ Facultad de Medicina Pontificia Universidad Javeriana Bogotá Colombia; ^6^ Fundación HOMI‐Hospital Pediátrico la Misericordia Unidad de Oncología y Hematología Pediátrica Bogotá Colombia; ^7^ Departamento de Pediatría Universidad Nacional de Colombia Bogotá Colombia; ^8^ Instituto Nacional de Cancerología Unidad de Oncología Pediátrica Bogotá Colombia; ^9^ Departamento de Pediatría Universidad del Valle Cali Colombia; ^10^ Hospital Universitario Fundación Valle del Lili Unidad de Oncología y Hematología Pediátrica Cali Colombia; ^11^ Clínica las Américas Auna, Instituto de Cancerología Las Américas Unidad de Oncología y Hematología Pediátrica Medellín Colombia; ^12^ Clínica Blas de Lezo Unidad de Oncología y Hematología Pediátrica Cartagena Colombia; ^13^ Division of Pediatric Hematology/Oncology, Department of Pediatrics University of California San Diego/Rady Children's Hospital San Diego San Diego California USA; ^14^ Population Sciences, Disparities and Community Engagement University of California San Diego Moores Cancer Center La Jolla California USA; ^15^ Dissemination and Implementation Science Center University of California San Diego Altman Clinical and Translational Research Institute La Jolla California USA

**Keywords:** bone neoplasms, COVID‐19, epidemiological monitoring, hospital, mortality, neoplasms, oncology service, pediatrics, retinoblastoma

## Abstract

**Background:**

Measures to control COVID‐19 transmission disrupted childhood cancer care. Data on the effects of the COVID‐19 pandemic on childhood cancer mortality are lacking. This study describes the impact of the pandemic on childhood cancer early‐mortality (≤ 24 months).

**Methods:**

A multicenter prospective cohort was conducted in 10 Colombian cities. Children with newly diagnosed cancer registered in the Childhood Cancer Clinical Outcomes Surveillance System (VIGICANCER) were included. Our primary outcome was cumulative mortality at 3, 6, 12, and 24 months. The exposed cohort (EC = March 25, 2020–December 31, 2021) was compared with a historic cohort (HC = January 1, 2017–March 24, 2020). Covariates included sociodemographics, place of residence, health insurance type, and tumor classification.

**Results:**

The cohort included 4124 children, comprised of 1627 children in the EC and 2497 children in the HC. Hematolymphoid, central nervous system, and extracranial solid tumors represented 57%, 15%, and 28% of patients, respectively. Participants' median age was 6.7 years (IQR, 3.2–11.3), 54% were male, 7% were Afro‐descendant, and 47% had public insurance. In the EC, the 6‐month and 24‐month mortality adjusted hazard ratio (aHR) in children with solid tumors was 1.7 (95% CI, 1.1–2.7) and 1.3 (95% CI, 1.0–1.7), respectively, and in children with bone tumors 4.0 (95% CI, 1.2–13.0) and 2.1 (95% CI, 1.2–3.6), respectively. These associations persisted after accounting for metastatic disease. Six‐month mortality aHRs for retinoblastoma, bone tumors, and soft tissue sarcomas due to progressive disease were 4.3 (95% CI, 1.3–14.5), 4.0 (95% CI, 1.4–11.3), and 5.4 (95% CI, 2.2–13.5), respectively. In the EC, the adjusted odds ratio (aOR) for metastatic solid tumors vs. nonmetastatic was 1.4 (95% CI, 1.0–1.8) and in children with retinoblastoma and public insurance the 24‐month mortality aHR was 4.9 (95% CI, 1.1–21.7).

**Conclusions:**

We observed increased early‐mortality for solid tumors, particularly bone tumors and retinoblastoma, likely attributed to more advanced‐stage presentation and loss of treatment effectiveness due to healthcare disruptions. Early‐mortality was higher in patients with public insurance, a vulnerable population that warrants attention.

## Graphical abstract

During the COVID‐19 pandemic, an increase in the risk of early‐mortality in children with solid malignant tumors was observed, specifically in bone tumors and retinoblastoma. Furthermore, patients were diagnosed at more advanced stages and the main cause of death was progressive disease. This study shows that the COVID‐19 pandemic contributed to advanced‐staged diagnoses and early‐mortality in children with solid tumors.

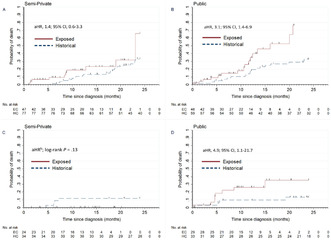

## 1 Introduction

During the COVID‐19 pandemic, healthcare was dramatically disrupted, including limited access to healthcare facilities, halted elective care, and postponed, rescheduled, or canceled outpatient visits and procedures [1, 2, 3, 4, 5, 6, 7]. The World Health Organization (WHO) defined levels of disruption of services in a healthcare system as *“completely disrupted,” “partially disrupted,” and “not disrupted”* if more than 50% of patients, 5%–50% of patients and < 5% of patients did not receive their planned care, respectively [1]. According to the WHO 2020 “Rapid Assessment [1] of the impact of the pandemic on healthcare services, disruptions varied from 39% in countries with low COVID‐19 infection burden to 56% in countries with high infection burden. In this assessment, Colombia was deemed with an intermediate transmission rate. However, after this report, and through March 2022, Colombia suffered four epidemic peaks, including a massive third peak in June 2021 with 33,000 cases, reaching a cumulative number of confirmed cases of 6 million and a mortality rate of 259 per 100,000 inhabitants, approximately 3.6 times the world's average [8, 9, 10, 11]. Latin American countries experienced complete or partial healthcare system disruptions, with the most impacted being those having healthcare expenditures of less than 7% of the gross domestic product [3, 11, 12, 13]. However, data on the disruption of cancer services in Colombia, a middle‐income country, are scarce.

Compared with the general population, higher mortality was reported in patients with cancer and concurrent COVID‐19 infection [3]. Moreover, disrupted access to timely care (e.g., diagnosis, surgery, radiotherapy, chemotherapy, and stem cell transplant) and unplanned treatment modifications constituted survival threats [14]. Furthermore, patients faced decision‐making challenges when considering interruptions in their cancer treatment versus risks of contracting the virus [15].

Although cancer care disruptions were reported by oncologists in Latin America, mainly during the first year of the COVID‐19 pandemic [3, 12, 16, 17], data lack on the effects of the COVID‐19 pandemic on childhood cancer mortality.

To address this knowledge gap, we analyzed data from the Childhood Cancer Clinical Outcomes Surveillance System (VIGICANCER) and investigated the effects of the pandemic in early‐mortality (≤ 24 months) and associated outcomes in children with cancer in Colombia.

## 2 Materials and Methods

### 2.1 Study Population and Setting

Colombia's population comprises 51 million, including 12 million children (< 15 years) [18]. VIGICANCER operates in 27 pediatric oncology units (POU) in 10 Colombian cities [19, 20], encompassing approximately 55% of all childhood cancer cases predicted to occur annually in Colombia [20].

VIGICANCER methodology has been described [19, 20]. VIGICANCER registers children and adolescents (< 19 years) newly diagnosed with cancer, and benign central nervous system (CNS) tumors (except for craniopharyngioma), receiving treatment in a participating POU.

VIGICANCER uses the International Agency for Cancer Research guide for diagnosis, with the most valid basis being microscopic (cytology and histology). If microscopic diagnosis is not feasible, methods like tumor markers (biochemical and immunologic) or clinical investigation are used. Clinical diagnosis alone, without any diagnostic technique, is insufficient for inclusion in VIGICANCER. Patients with a diagnosis by death certificate are accepted.

Informed consent was obtained from parents/legal guardians of eligible children after approvals by Institutional Review Boards at the Universidad del Valle and participating sites.

### 2.2 Cohort Definition

The exposed cohort (EC) included children diagnosed with cancer registered in VIGICANCER from March 25, 2020 (national COVID‐19 lockdown in Colombia), to December 31, 2021. We compared EC to a historical cohort (HC) from January 1, 2017, to March 24, 2020.

### 2.3 Outcome Measures and Follow‐Up

Our primary outcome was cumulative mortality at 3, 6, 12, and 24 months. Secondary outcomes were metastatic disease, relapse/progression, treatment abandonment, and cause of death. Early mortality (≤ 24 months) was classified as: (a) cancer‐related (due to relapse/progression), (b) treatment‐related, (c) unrelated to cancer diagnosis and/or treatment (during treatment or after completion), and (d) unknown.

Causes and timing of death were ascertained from medical records and centrally reviewed by VIGICANCER investigators (OR and VP), following VIGICANCER protocol definitions and based on information about remission status, timing of treatment receipt, and causes of death.

VIGICANCER defines treatment abandonment as nonmedical related delays in curative therapy for > 4 weeks due to the patient not returning for care [21]. VIGICANCER conducts active follow‐up every 3 months to collect information on outcomes [8, 9]. If a patient completes cancer treatment and can not be contacted after three attempts in a 6‐month period, lost to follow‐up is documented. For those lost to follow‐up, passive surveillance is conducted by accessing two separate governmental platforms to gather vital status.

### 2.4 Covariates

Covariates included age at diagnosis, sex, parent‐reported Afro‐descendant ethnicity, health insurance type, residing in a city with a POU, and tumor classification.

Colombia's health insurance system covers 85% of inhabitants under two main plans: semiprivate (employed/self‐employed) and public (unemployed, informal/low‐income self‐employed) [22, 23]. Ten percent of inhabitants have private/government plans and approximately 4% are uninsured.

Participants were classified as residing (≥ 6 months before cancer diagnosis), or not, in a city with a POU (large cities were defined as ≥ 100 cancer cases/year, small < 100 cancer cases/year).

Tumor classification was based on the International Classification of Diseases for Oncology (ICCC‐3) [24], and hematolymphoid tumors per the WHO 2008 classification [25].

### 2.5 Statistical Analysis

We estimated relative frequencies and central tendency measures and examined sociodemographics by cohort and tumor classification. For solid tumors, we adjusted the risk of metastatic disease at diagnosis between cohorts (EC vs. HC), estimating adjusted odds ratio (aOR) using conditional (by city) logistic multivariable regression and included covariates of interest: age, sex, ethnicity, insurance type, and residence. We evaluated collinearity and performed the Hosmer–Lemeshow goodness‐of‐fit test for each model.

For survival analyses, we estimated time from diagnosis date to event of interest date or last contact (for those without an event). The median time between the diagnosis of a case to its registration in VIGICANCER was 91 days (IQR 29–231; EC = 103 vs. HC = 79 days; *p* = 0.01). Analyses cutoff date was July 27, 2022. We treated treatment abandonment as informed censorship if the vital status was not verified and assigned an event at the treatment abandonment date. Patients were censored in the analysis if lost to follow‐up or if transferred to a POU not affiliated with VIGICANCER and if their vital status was not determined through passive surveillance.

We calculated cumulative mortality at 3, 6, 12, and 24 months by using Kaplan–Meier. To estimate the effect of the exposure (COVID‐19 pandemic), we constructed proportional hazards Cox's regression models stratified by city, using covariates of interest to calculate adjusted hazard rate ratios (aHR). Metastatic disease was considered as an early‐mortality mediator and not as confounding factor. We estimated changes in aHRs adjusted and unadjusted for metastatic disease in multivariable models and compared model coefficients to determine effects of metastatic status. We evaluated proportional hazards assumptions for each model graphically and with the Schoenfeld residual test. We also conducted competing risk analysis for cause of death. We estimated cumulative risks of death from progressive disease, and adjusted competing cause‐specific hazard rate ratios (csHR), including other causes of death as competing risks. Missing data were managed with appropriate measures. Data management was conducted using REDCap [26, 27] hosted at Cali's Population‐Based Cancer Registry (Universidad del Valle).

We used STATA v.17.0, estimated 95% confidence intervals (CI) and considered a two‐tailed *p* value < 0.05 as significant.

## 3 Results

### 3.1 Patient Characteristics

During the study period, VIGICANCER included 4124 children, 1627 (39%) in the EC and 2497 (61%) in the HC. We excluded 199 patients who did not meet inclusion criteria. Eighteen parents (0.44%) of eligible patients declined participation.

**FIGURE 1:**
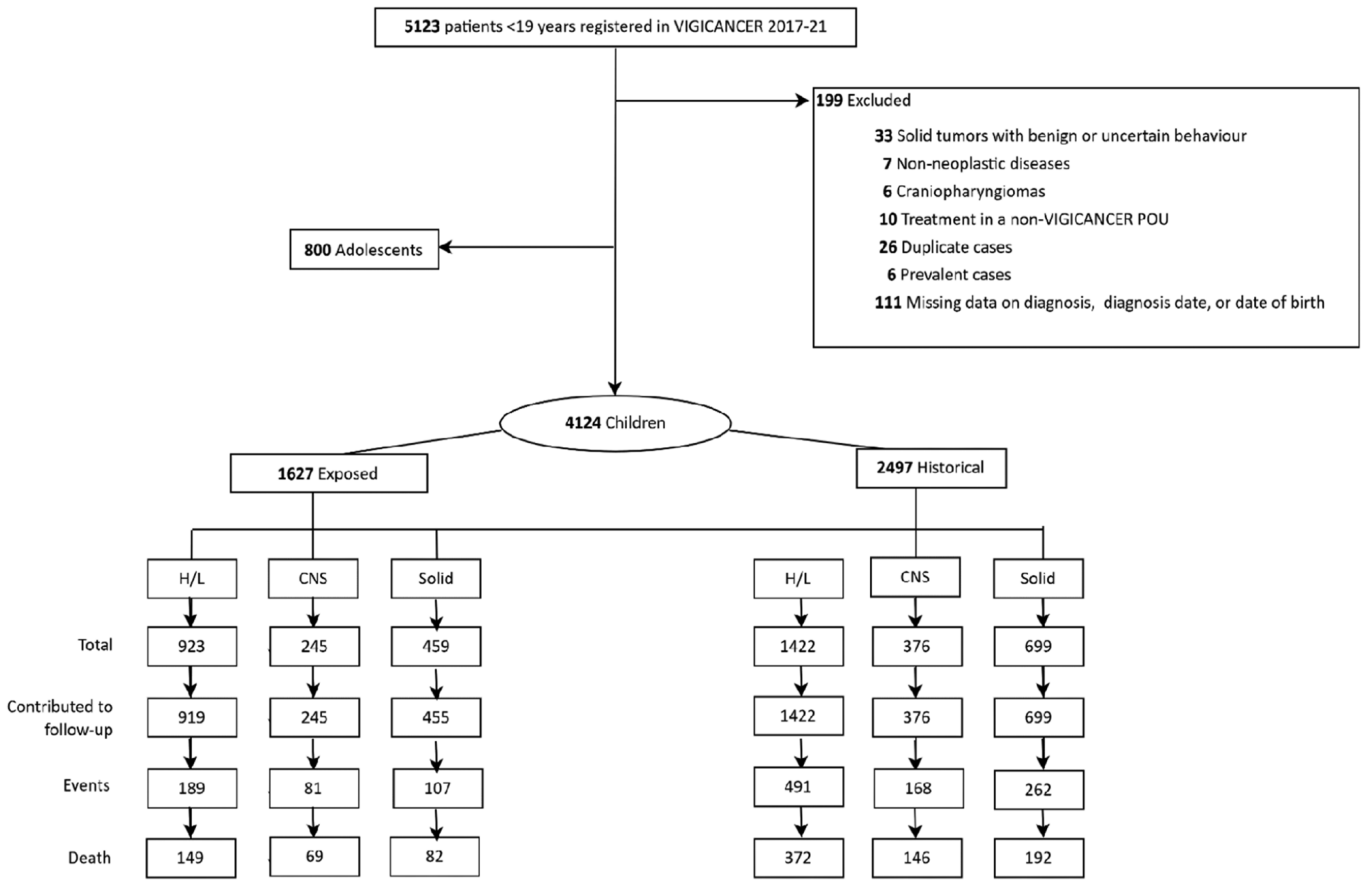
Flowchart of study participants by cohort. CNS, central nervous system tumors; H/L, hematolymphoid tumors; Solid, solid tumors; POU, pediatric oncology unit. Exposed cohort, January 1, 2017, to March 24, 2020; historical cohort, March 25, 2020, to December 31, 2021). Children's age range was 0.2–14.9 years. The flowchart illustrates the distribution of patients in the exposed and historical cohort, the number of events (relapse, death, or treatment abandonment where no further information was available), and deaths.

Follow‐up information was available in 4116 (99%) of cases. Figure 1 shows the study flowchart by cohort. Hematolymphoid, CNS, and solid tumors represented 57% (*n* = 2345), 15% (*n* = 621), and 28% (*n* = 1158) of patients, respectively. Table 1 summarizes participants' characteristics. Table S1 shows their distribution by ICCC‐3 tumor group. Participants' median age was 6.7 years (IQR, 3.2–11.3), 54% (*n* = 2235) were male, 7% (*n* = 266) were Afro‐descendant (EC 6% vs. HC 7%; *p* = 0.07), 40% (*n* = 2471) resided in a city with POU (EC 42% vs. HC 38%; *p* = 0.01), 47% (*n* = 1924) had public insurance (EC 49% vs. HC 45%; *p* = 0.03), and 3% (*n* = 114) were uninsured (EC 1% vs. HC 4%; *p* < 0.01).

**TABLE 1:** Patient baseline characteristics by cohort^a^.

Characteristics^b^	Exposed cohort		Historical cohort		Total		*p*
	*N*	(%)	*n*	(%)	*n*	(%)	
Age group (years)							
< 1	87	(5)	151	(6)	238	(6)	0.72
1–4	534	(33)	818	(33)	1352	(33)	
5–9	458	(28)	716	(29)	1174	(28)	
10–14	548	(34)	812	(33)	1360	(33)	
Sex							
Male	856	(53)	1379	(55)	2235	(54)	0.10
Female	771	(47)	1118	(45)	1889	(46)	
Afro‐descendant ethnicity							
Yes	91	(6)	175	(7)	266	(6)	0.20
No	1525	(94)	2305	(92)	3830	(93)	
Missing	11	(1)	17	(1)	28	(1)	
Residing in a city with a POU							
Yes	681	(42)	951	(38)	1632	(40)	0.01
No	934	(57)	1537	(62)	2471	(60)	
Missing	12	(1)	9	(0)	21	(1)	
Cities (new cancer cases/year)							
> 100	1225	(75)	1847	(74)	3072	(75)	0.34
< 100	402	(25)	650	(26)	1052	(26)	
Health insurance affiliation							
Public	795	(49)	1129	(45)	1924	(47)	< 0.001
Semiprivate	717	(44)	1092	(44)	1809	(44)	
Private insurance/special plans^c^	92	(6)	170	(7)	262	(6)	
Uninsured	19	(1)	95	(4)	114	(3)	
Missing	4	(0)	11	(0)	15	(0)	
Tumor group							
Hematolymphoid	923	(57)	1422	(57)	2345	(57)	0.99
CNS tumors	245	(15)	376	(15)	621	(15)	
Solid tumors	459	(28)	699	(28)	1158	(28)	

Abbreviations: CNS, central nervous system; POU, pediatric oncology unit.

^a^ Exposed cohort, January 1, 2017, to March 24, 2020; historical cohort, March 25, 2020, to December 31, 2021.

^b^ 4124 is the total number of participants unless otherwise indicated. Of note, the sum of some of the totals may not be exactly 100% due to rounding.

^c^ Special plans include special regimens for certain government groups, such as police, military, and state‐owned organizations.

### 3.2 Metastatic Disease at Diagnosis in Children with Solid Tumors by Cohort, Insurance Type, and Residence (Table S2)

One‐third (*n* = 331) of children with solid tumors (*n* = 984) had metastatic disease at diagnosis (EC 37% vs. HC 31%; aOR, 1.4; 95% CI, 1.0–1.8). Fifteen percent of patients (*n* = 174) were not included in this analysis because of data missing regarding metastatic status. In children < 10 years (*n* = 636), Afro‐descendants (*n* = 71), and those not residing in a city with POU (*n* = 396), metastatic disease was present in the EC vs. the HC in 35% vs. 28% (aOR, 1.5; 95% CI, 1.0–2.1), 54% vs. 26% (aOR, 3.4; 95% CI, 1.0–12.1), and 42% vs. HC 32% (aOR, 1.7; 95% CI, 1.2–2.4), respectively. In children with public insurance (*n* = 422), metastatic disease was present in the EC vs. the HC in 40% vs. 35% (aOR, 1.2; 95% CI, 0.8–1.8); whereas in children with semiprivate insurance (*n* = 468), it was 37% vs. 26% (aOR, 1.7; 95% CI, 1.1–2.6), respectively.

### 3.3 Metastatic Disease at Diagnosis by Tumor Group and Insurance Type (Table 2)

**TABLE 2:** Association (aOR) between the metastatic disease by solid tumor ICCC‐3 group and health insurance^a^.

Solid tumors		C^b^	Public					Semiprivate					Total				
			Metastatic			aOR (95% CI)		Metastatic			aOR (95% CI)		Metastatic			aOR (95% CI)	
			*m*	*n*	(%)			*m*	*n*	(%)			*m*	*n*	(%)		
IV.	Neuroblastoma	E	9	15	(60)	1.2	(0.2–7.0)	6	14	(43)	0.9	(0.2–4.1)	15	30	(50)	0.9	(0.3–2.5)
		H	13	21	(62)	[Reference]		12	34	(35)	[Reference]		31	68	(46)	[Reference]	
V.	Retinoblastoma	E	16	29	(55)	2.4	(0.5–12.0)	12	23	(52)	4	(0.3–59.3)	30	56	(54)	3.7	(1.1–12.1)
		H	8	29	(28)	[Reference]		7	28	(25)	[Reference]		19	67	(28)	[Reference]	
VI.	Renal tumors	E	6	28	(21)	0.5	(0.1–2.0)	2	30	(7)	0.3	(0.1–1.7)	9	61	(15)	0.5	(0.2–1.3)
		H	11	45	(24)	[Reference]		11	51	(22)	[Reference]		22	107	(21)	[Reference]	
VII.	Hepatic tumors	E	3	9	(33)	0.7	(0.1–7.9)	3	11	(27)	0.9	(0.1–8.1)	6	22	(27)	1.1	(0.3–4.7)
		H	10	19	(53)	[Reference]		5	21	(24)	[Reference]		16	43	(37)	[Reference]	
VIII.	Malignant bone tumors	E	18	34	(53)	3.4	(1.1–10.6)	20	42	(48)	1.4	(0.6–3.4)	40	85	(47)	1.8	(1.0–3.2)
		H	18	53	(34)	[Reference]		22	65	(34)	[Reference]		47	128	(37)	[Reference]	
IX.	Soft tissue sarcomas	E	4	22	(18)	0.5	(0.1–2.1)	9	24	(38)	12	(1.2–113.8)	14	47	(30)	1.7	(0.7–4.4)
		H	14	38	(37)	[Reference]		4	36	(11)	[Reference]		24	87	(28)	[Reference]	
X.	Germ cell neoplasms	E	7	25	(28)	2.8	(0.6–12.9)	6	20	(30)	2	(0.4–8.7)	13	50	(26)	2.4	(0.9–6.7)
		H	3	28	(11)	[Reference]		6	36	(17)	[Reference]		9	69	(13)	[Reference]	
XI.	Other epithelial and melanomas	E	5	11	(45)	0.2	(0.0–4.7)	7	10	(70)	7.9	(0.3–218.4)	12	21	(57)	1.4	(0.3–6.5)
		H	9	15	(60)	[Reference]		10	21	(48)	[Reference]		23	40	(58)	[Reference]	
Total		E	69	174	(40)	1.2	(0.8–1.8)	65	176	(37)	1.7	(1.1–2.6)	140	375	(37)	1.4	(1.0–1.8)
		H	86	248	(35)	[Reference]		77	292	(26)	[Reference]		191	609	(31)	[Reference]	
All solid tumors^c^		—	155	422	(37)	—		142	468	(30)	—		331	984	(34)	—	

Abbreviations: aOR, adjusted odds ratio estimated by conditional logistic regression, adjusted for age, sex, Afro‐descendant ethnicity, and place of residence, grouped by city, with the historical cohort as reference; C, cohort; E, exposed; H, historical; ICCC, international classification of childhood cancer; *m*, number of participants with metastatic disease at diagnosis in each stratum; *n*, total number of participants with information in each stratum.

^a^ All the categories of health insurance are included in the total column. Private/special plans and uninsured categories are not shown due to their low number of participants.

^b^ The exposed cohort (March 25, 2020, to December 31, 2021) was compared with the historical cohort (January 1, 2017, to March 24, 2020) as defined in the methods.

^c^ This row shows the total patients included, 15% of patients (*n* = 174) were not included in the analysis due to missing data regarding metastatic status.

Out of 213 children diagnosed with bone tumors (with information about metastatic disease), 87 (41%) had metastatic disease (EC 47% vs. HC 37%; aOR, 1.8; 95% CI, 1.0–3.2); 41% in those with public insurance (*n* = 87; EC 53% vs. HC 34%; aOR, 3.4; 95% CI, 1.1–10.6), and 39% in those with semiprivate insurance (*n* = 107; EC 48% vs. HC 34%; aOR, 1.4; 95% CI, 0.6–3.4). Metastatic disease in children with retinoblastoma (*n* = 49/123) was 40% (EC 54% vs. HC 28%; aOR, 3.7; 95% CI, 1.1–12.1). In children with retinoblastoma and public insurance (*n* = 58), metastatic disease was present in 41% (EC 55% vs. HC 28%; aOR, 2.4; 95% CI, 0.5–12.0), and in those with semiprivate insurance (*n* = 51), it was 37% (EC 52% vs. HC 25%; aOR, 4.0; 95% CI, 0.3–59.3).

Retinoblastoma was diagnosed above 1 year of age in 83% of cases in the EC and in 60% of cases in the HC (aOR, 3.3; 95% CI, 1.4–7.8).

### 3.4 Early‐Mortality

The cumulative mortality for the entire cohort at 3, 6, 12, and 24 months was 7% (95% CI, 7%–8%), 11% (95% CI, 10%–12%), 18% (95% CI, 17%–20%), and 27% (95% CI, 25%–28%), respectively. At the 24‐month follow‐up, the EC showed an aHR 1.1 (95% CI, 0.9–1.3) and in patients with public insurance an aHR 1.3 (95% CI, 1.1–1.5).

### 3.5 Six‐Month Mortality by Cohort and Tumor Group (Table 3)

**TABLE 3:** Adjusted mortality hazards rate ratio by tumor group (ICCC) at 6 and 24 months.

Tumor group^a^		C	*n*	6 months				24 months			
				*D*	*M* (%)	aHR	(95% CI)	*D*	*M* (%)	aHR	(95% CI)
I.	Leukemias	E	736	80	(11)	1.0	(0.7–1.3)	128	(27)	1.0	(0.8–1.2)
		H	1132	138	(12)	[Reference]		287	(26)	[Reference]	
II.	Lymphomas	E	183	11	(6)	1.0	(0.4–2.1)	21	(15)	1.4	(0.8–2.5)
		H	290	18	(6)	[Reference]		36	(13)	[Reference]	
III.	Primary CNS tumors	E	245	31	(16)	1.0	(0.6–1.5)	68	(42)	1.1	(0.8–1.5
		H	376	57	(15)	[Reference]		140	(38)	[Reference]	
IV.	Neuroblastoma	E	40	5	(13)	1.1	(0.2–5.1)	9	(32)	0.8	(0.3–2.1)
		H	79	8	(10)	[Reference]		28	(38)	[Reference]	
V.	Retinoblastoma	E	57	5	(10)	2.1	(0.5–8.0)	8	(19)	2.0	(0.6–6.3)
		H	77	4	(5)	[Reference]		9	(12)	[Reference]	
VI.	Renal tumors	E	76	4	(5)	1.1	(0.3–4.1)	8	(31)	1.1	(0.4–2.9)
		H	117	6	(5)	[Reference]		15	(14)	[Reference]	
VII.	Hepatic tumors	E	27	1	(4)	0.1	(0.0–1.2)	2	(12)	0.1	(0.0–0.6)
		H	45	10	(22)	[Reference]		17	(38)	[Reference]	
VIII.	Malignant bone tumors^b^	E	105	11	(11)	4.0	(1.2–13.0)	27	(65)	2.1	(1.2–3.6)
		H	148	4	(3)	[Reference]		45	(33)	[Reference]	
IX.	Soft tissue sarcomas	E	55	6	(11)	4.1	(0.8–20.3)	13	(44)	1.3	(0.6–2.8)
		H	101	4	(4)	[Reference]		36	(38)	[Reference]	
X.	Germ cell tumors	E	61	6	(10)	3.5	(0.8–15.8)	8	(18)	2.4	(0.7–7.5)
		H	81	3	(4)	[Reference]		6	(8)	[Reference]	
XI.	Other epithelial and melanomas	E	30	3	(10)	5.0	(0.4–60.8)	5	(36)	3.8	(0.6–22.4)
		H	47	2	(5)	[Reference]		7	(16)	[Reference]	
Total for solid tumors^c^		E	455	42	(10)	1.7	(1.1–2.6)	81	(35)	1.3	(1.0–1.7)
		H	699	42	(6)	[Reference]		164	(25)	[Reference]	
Total for all the tumor groups		E	1619	170	(11)	1.1	(0.9–1.3)	298	(30)	1.1	(0.9–1.3)
		H	2497	255	(10)	[Reference]		627	(26)	[Reference]	

Abbreviations: aHR, adjusted hazard rate ratio estimated by proportional hazards Cox's multivariable regression, adjusted for age, sex, Afro‐descendant ethnicity, place of residence, and health insurance type, stratified by city, with historical cohort as reference; C, cohort; CNS, central nervous system; *D*, number of deaths that occurred within 6 and 24 months after diagnosis; E, exposed; H, historical; ICCC, international classification of childhood cancer; *M*, cumulative mortality as percentage; *n*, number of patients contributing to follow‐up.

^a^ Group XII is not shown due to their low number of participants.

^b^ For malignant bone tumors 24‐month mortality, including metastatic status in the regression model changed de β coefficient in 7%.

^c^ For 6‐ and 24‐month mortality, the β coefficients with and without the metastatic status variable in the model was 0.43 vs. 0.52 (−21% difference), and of 0.19 vs. 0.26 (−37% difference), respectively.

Six‐month cumulative mortality in the EC vs. HC for children with hematolymphoid, CNS, and solid tumors was 10% (95% CI, 8%–12%) vs. 11% (95% CI, 10%–13%), 16% (95% CI, 12%–21%) vs. 15% (95% CI, 12%–19%), and 10% (95% CI, 7%–13%) vs. 6% (95% CI, 5%–8%), respectively. The 6‐month mortality aHR between cohorts in children with solid tumors was 1.7 (95% CI, 1.1–2.6), and, when adjusted for metastatic status (including missing values), was 1.5 (95% CI, 1.0–2.4).

In children with solid metastatic tumors, 6‐month mortality in the EC vs. HC was 21% (95% CI, 15%–29%) vs. 12% (95% CI, 8%–18%), respectively; aHR 2.0 (95% CI, 1.1–3.6). In patients with public insurance and solid tumors, the 6‐month mortality in the EC vs. HC was 13% (95% CI, 9%–18%) vs. 9% (95% CI, 7%–14%), respectively; aHR 1.5 (95% CI, 0.9–2.6).

### 3.6 Twenty‐Four‐Month Mortality by Cohort and Tumor Group (Table 3)

Twenty‐four‐month cumulative mortality in the EC vs. HC for children with hematolymphoid, CNS, and solid tumors was 25% (95% CI, 20%–31%) vs. 24% (95% CI, 21%–26%), 42% (95% CI, 33%–52%) vs. 38% (95% CI, 34%–44%), and 35% (95% CI, 27%–45%) vs. 25% (95% CI, 22%–29%), respectively. The aHR at 24 months in exposed children with solid tumors was 1.3 (95% CI, 1.0–1.7), and 1.2 (95% CI, 0.9–1.6) when adjusted for metastatic status.

In children with public insurance and solid tumors, the 24‐month mortality in the EC vs. HC was 47% (95% CI, 32%–64%) vs. 28% (95% CI, 23%–34%); aHR 1.5 (95% CI, 1.0–2.1).

### 3.7 Early‐Mortality in Malignant Bone Tumors (ICCC Group VIII, Figure 2)

**FIGURE 2:**
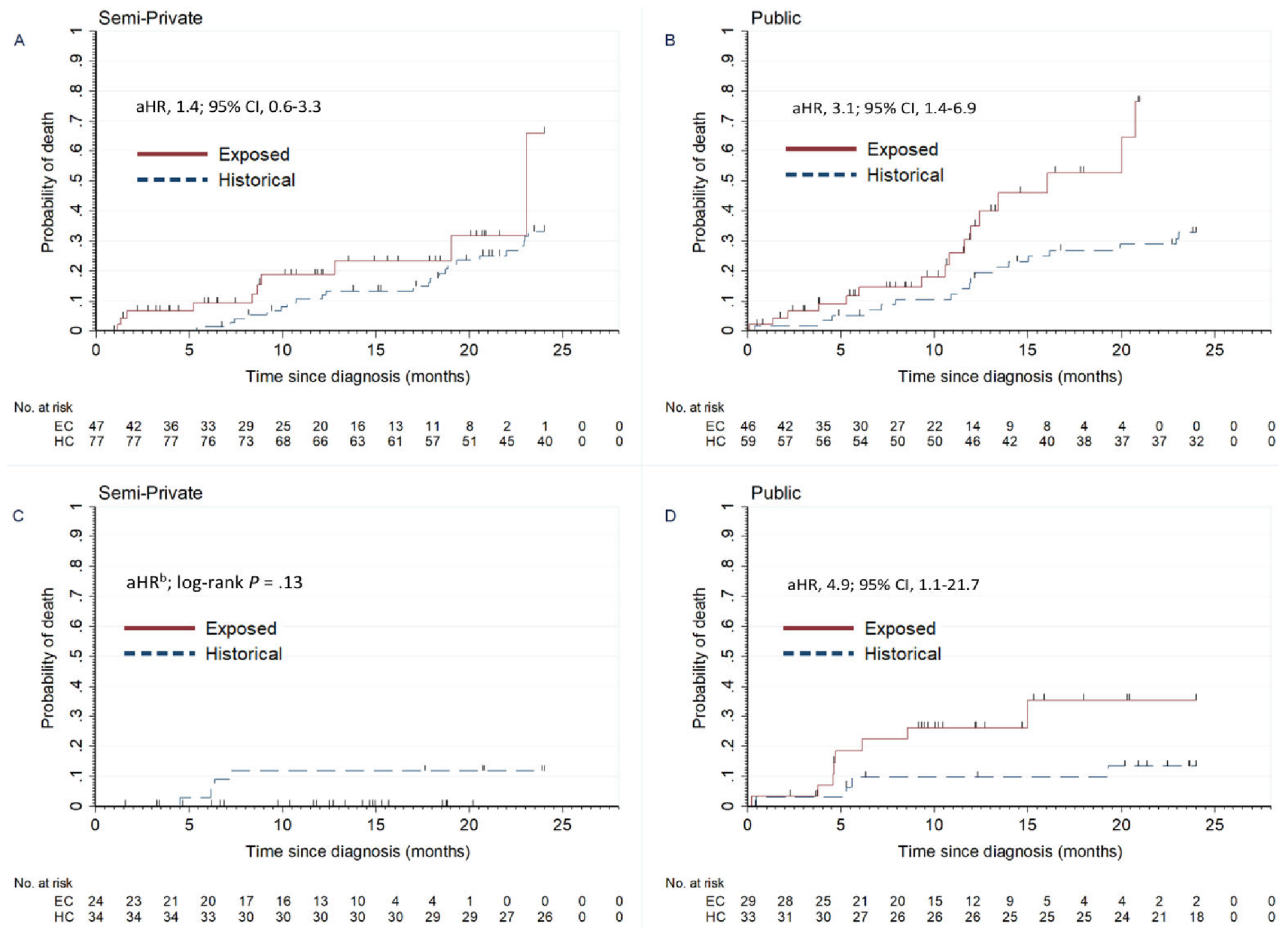
Cumulative mortality of patients with malignant bone tumors and retinoblastoma by health insurance group between cohorts. (A) In children with bone tumors and semiprivate insurance, 24‐month mortality was 1.4 times higher in the exposed cohort compared with the historical cohort (aHR, 1.4; 95% CI, 0.6–3.3). (B) In children with bone tumors and public insurance, mortality at 24 months was 3.1 times higher in the exposed cohort compared with the historical cohort (aHR, 3.1; 95% CI, 1.4–6.9). (C) In children with retinoblastoma and semiprivate insurance, no deaths were registered in the exposed cohort follow‐up. (*D*) In children with retinoblastoma and public insurance, mortality at 24 months was 4.9 times higher in the exposed cohort compared with the historical cohort (aHR, 4.9; 95% CI, 1.1–2.7). Including metastatic status in the regression model with 24 months exit time changed the β coefficient by 7%. *Children's age range was 0.2–14.9 years.

In children with bone tumors (*n* = 253), the cumulative mortality in the EC (*n* = 105) vs. the HC (*n* = 148) at 3, 6, 12, and 24 months was 6% (95% CI, 3%–13%) vs. 1% (95% CI, 0%–5%), with aHR of 8.0 (95% CI, 0.9–69.4); 12% (95% CI, 7%–20%), vs. 3% (95% CI, 1%–7%), with aHR of 4.0 (95% CI, 1.2–13.0); 24% (95% CI, 16%–36%) vs. 13% (95% CI, 9%–20%), with aHR of 2.2 (95% CI, 1.1–4.3); and 65% (95% CI, 37%–91%) vs. 33% (95% CI, 26%–42%), with aHR of 2.1 (95% CI, 1.2–3.6), respectively. In children with bone tumors and public insurance, aHR was 2.6 (95% CI, 1.1–6.3) when adjusted for metastatic disease.

### 3.8 Early‐Mortality in Retinoblastoma by Insurance Type

In children with retinoblastoma (*n* = 134), no deaths were registered in those with private or semiprivate insurance in the EC (*n* = 66). Cumulative mortality for children with public insurance (*n* = 62) is shown in Figure 2D. The 24‐month mortality aHR in children with retinoblastoma and public insurance was 5.3 (95% CI, 1.3–21.5).

### 3.9 Progressive Disease as Cause of Death

Patients with solid tumors had a 3, 6, 12, and 24‐month cumulative mortality because of progressive disease of 2% (EC 2% vs. HC 1%; csHR 1.6; 95% CI, 1.1–2.3), 4% (EC 4% vs. HC 2%; aHR 1.9; 95% CI, 1.4–2.5), 7% (EC 8% vs. HC 7%; csHR 1.2; 95% CI, 0.8–1.8), and 11% (EC 12% vs. HC 11%; csHR 1.1; 95% CI, 0.8–1.6), respectively. Including metastatic disease status in the 3‐ and 6‐month competitive risks multivariable models, csHR (EC vs. HC) was 1.4 (95% CI, 1.0–2.0) and 1.7 (95% CI, 1.2–2.3), respectively.

Table 4 shows csHR for 6‐month progressive disease mortality by tumor group. Six‐month csHR for retinoblastoma, malignant bone tumors, and soft tissue sarcomas was 4.3 (95% CI, 1.3–14.5), 4.0 (95% CI, 1.4–11.3), and 5.4 (95% CI, 2.2–13.5), respectively. Within the public insurance, the 6‐month csHR for children with retinoblastoma was 7.6 (95% CI, 2.1–27.5).

**TABLE 4:** Six‐month progressive disease mortality hazards rate ratio by ICCC tumor group.

Tumor group^a^	*N*	PD	OD	csHR	(95% CI)
I. Leukemias	1856	40	177	1.2	(0.3–3.9)
II. Lymphomas	470	19	10	0.8	(0.4–1.5)
III. Primary CNS tumors	618	77	17	1.0	(0.7–1.4)
IV. Neuroblastoma	119	4	9	0.8	(0.1–5.8)
V. Retinoblastoma	134	6	3	4.3	(1.3–14.5)
VI.^b^ Renal tumors	193	9	1	1.4	(0.4–5.0)
VII. Hepatic tumors	72	6	5	0.3	(0.0–3.7)
VIII. Malignant bone tumors	252	10	5	4.0	(1.4–11.3)
IX. Soft tissue sarcomas	156	9	1	5.4	(2.2–13.5)
X. Germ cell tumors	142	8	1	2.4	(0.7–7.8)
XI. Other epithelial and melanomas	77	5	0	2.2	(0.1–39.0)
Total for solid tumors	1153	59	25	1.9	(1.4–2.5)
Total	4097	195	229	1.2	(0.8–1.8)

Abbreviations: CNS, central nervous system; csHR, adjusted cause‐specific (progressive disease) mortality hazard rate ratio estimated by competing risks multivariable regression, adjusted for age, sex, Afro‐descendant ethnicity, place of residence, and health insurance type, grouped by city, with historical cohort as reference; ICCC, international classification of childhood cancer; *N*, total number of participants included in the regression models; OD, number of other causes of death that occurred within 6 months after diagnosis; PD, number of progressive deaths that occurred within 6 months after diagnosis.

^a^ Group XII is not shown due to their low number of participants.

^b^ csHR for groups VI and XI could not be adjusted by all the covariates of interest; they were adjusted by age, sex, Afro‐descendant ethnicity, and place of residence, grouped by city.

### 3.10 Relapse and Treatment Abandonment

The entire cohort cumulative incidence of relapse (*n* = 405) at 6, 12, and 24 months was 2% (95% CI, 2%–3%), 7% (95% CI, 6%–8%), and 15% (95% CI, 14%–17%), respectively, whereas the cumulative incidence of relapse in EC vs. HC was 11% vs. 16%, respectively (aHR = 0.7; 95% CI, 0.7–0.9). The 12‐month cumulative incidence of treatment abandonment (*n* = 157) was 4% (95% CI, 4%–5%; EC 3% vs. HC 5%).

## 4 Discussion

We observed that deaths in children with solid tumors largely contributed to the excess in childhood cancer early‐mortality during the COVID‐19 pandemic, with a 70% increase in mortality risk at 6 months. We did not find a similar increase in hematolymphoid or CNS tumors. Children with bone tumors had a consistently higher risk of death. Six‐month mortality risk was four times greater than pre‐pandemic, and the risk remained twice as high at 24 months. Furthermore, mortality risk for children with bone tumors in the EC was higher in patients with public insurance, compared with patients with semiprivate insurance, suggesting disparate outcomes by insurance type, which is consistent with prior research [28, 29, 30, 31, 32]. Similarly, children with retinoblastoma and public insurance had a five‐fold greater risk of death at 24 months.

The increased risk of early‐mortality in children with solid tumors in the EC was mainly due to progressive disease. Children with solid tumors in the EC had a twofold increase in progressive disease mortality risk at 6 months, which persisted after adjusting for metastatic disease. Across solid tumor groups, we found that the increased risk of death in the EC attributable to progressive disease was significant for retinoblastoma, bone tumors, and soft tissue sarcomas.

Our findings suggest that the isolation and lockdown measures during the pandemic led to delays in cancer diagnosis and resulted in advance‐stage presentations. The impact of these delays was evidenced by a 40% increase in metastatic disease at presentation in the EC compared with HC. Moreover, those with semiprivate insurance had a twofold higher risk of metastatic disease in the EC compared with the HC. Relatedly, we observed a threefold increase in the frequency of retinoblastoma diagnosed in children older than 1 year of age in the EC. This finding is consistent with reports in LMIC showing that failures in early detection of retinoblastoma lead to late diagnosis at older age [33]. This shift in retinoblastoma diagnostic age was evident in larger cities but not in smaller ones.

Our results underscore that treatment interruptions during the pandemic likely decreased treatment effectiveness. The most plausible explanation for progressive disease‐related deaths in patients without metastasis is loss of treatment effectiveness, attributed to reduced doses of chemotherapy, longer intervals between chemotherapy cycles, and delayed access to surgery and radiotherapy [34]. Timely access to surgical interventions is crucial to improve survival in patients with solid tumors [35, 36, 37]. The pandemic severely affected access to and availability of timely surgical services and caused significant disruptions in operating rooms and intensive care units, resulting in substantial scheduling delays and procedure cancelations [38]. A study of 169 patients from 30 Indian centers showed that 22%–39% of patients faced surgery, radiotherapy, and chemotherapy delivery delays and/or interruptions, and that 23% of patients with sarcoma received decreased‐intensity neoadjuvant chemotherapy during the pandemic [39, 40, 41]. Our results contribute to the scant literature on mortality associated with disruption of care because of the pandemic.

The disparities in outcomes by COVID‐19 pandemic exposure, insurance type, Afro‐descendant ethnicity, and residence, suggest that public health initiatives to mitigate the effects of COVID‐19 affected the population disproportionally. As reported by others, the COVID‐19 pandemic widened the already known inequities in access to healthcare in marginalized groups [42]. Our findings illustrate how underserved individuals were affected by the consequences of public health measures to control COVID‐19 transmission. In our study, the impact of the pandemic appeared to be an “urban phenomenon” as exposed patients residing in larger cities or those with semiprivate insurance seemed to have an increased risk of metastatic disease. Urban residents may have implemented more rigorous isolation protocols, not only in compliance with public health directives but also due to concerns about virus transmission [43, 44]. Moreover, access to care could have been affected by a digital divide [45, 46]. As in many other LMIC, Colombia's digital connectivity is unequal across regions because of inadequate infrastructure in rural areas, limited digital literacy, and expensive internet and phone fees [47, 48, 49]. This could have led to restricted access to telemedicine, which rapidly increased during the pandemic [50, 51, 52]. Moreover, shifting to virtual consultations in LMIC could also contribute to delayed diagnosis [53], as at the beginning of the pandemic, telemedicine was uncommon in resource‐constrained settings and most physicians had not received training in virtual physical examination techniques, hindering their ability to identify early signs of cancer [54].

Although our cohort included a large sample, it has limitations. Defining the exposure timeframe was challenging, as we used the start and end of the “pandemic period” per the government's determination, and exposure misclassification may have occurred. Similarly, our sample size could be viewed as limited because of the relatively low incidence of childhood cancer, the under‐registration of CNS tumors expected in the catchment area, and the timeframe restrictions implicit in the exposure. The follow‐up of patients in our cohort was 24 months, precluding the evaluation of the long‐term effects of the COVID‐19 pandemic exposure on survival. A better understanding of the pandemic effects on survival due to loss of treatment effectiveness will require longer follow‐up, particularly in hematological malignancies. We found an unexpected decrease in treatment abandonment and relapse that could be explained by interruptions in VIGICANCER's operations and patient registration. The pandemic lockdown and subsequent restrictions severely constrained data collection; therefore, we may not have accurately registered treatment abandonment. While relapses are easier to detect, they could have been underreported because of data collection disruptions. Early deaths due to tumor progression acted as a competing event for treatment abandonment and relapse, probably contributing to the decrease in occurrence of these two events. It is possible that advanced‐stage tumors could have been considered incurable by non‐oncology physicians (e.g., pediatricians and surgeons). These patients may not have been referred to a POU during the pandemic and, therefore, might not have been registered by VIGICANCER. This potential bias could have diminished the effect we observed. However, we do not consider bias because of attrition being likely because the follow‐up was short (24 months), and we had low proportion of lost to follow‐up cases (< 5%). Overall, we have no evidence that our findings could be explained by changes in referral patterns during the pandemic or in procedures to track patients. Lastly, we acknowledge possible uncontrolled confounding factors that could account for some observed associations.

## 5 Conclusions

In conclusion, our findings support an increase in early‐mortality for solid tumors during the COVID‐19 pandemic, likely due to the occurrence of more advanced‐stage tumors at diagnosis and a short‐term loss of treatment effectiveness. However, the effect was not uniform among all solid tumors. The impact of the pandemic on timely diagnosis of childhood cancer and effective treatment was disproportionate in children from disadvantaged backgrounds (e.g., public insurance). Mechanisms contributing to the increased early‐mortality in children with solid tumors in Colombia during the pandemic are multifactorial. Our results highlight barriers to healthcare access and disparities in vulnerable communities most affected by the pandemic in LMIC that warrant further investigation. Continued monitoring of this cohort is necessary to assess the long‐term effects of the COVID‐19 pandemic on survival of children with cancer. Understanding the consequences of the recent pandemic on childhood cancer mortality in LMIC will shed light on barriers in healthcare systems in both LMIC and high‐income countries amenable to intervention, and will aid public health policy and preparedness for future health crises.

### Précis

This study shows that the COVID‐19 pandemic contributed to advanced‐staged diagnoses and increased early‐mortality in children with solid tumors, specifically in bone tumors and retinoblastoma. Furthermore, the main cause of death was progressive disease, likely attributed to loss of treatment effectiveness due to healthcare disruptions.

## Author Contributions

**Oscar Ramirez:** conceptualization (lead), data curation (lead), formal analysis (lead), funding acquisition (lead), investigation (lead), methodology (lead), project administration (lead), resources (equal), supervision (equal), writing – original draft (lead), writing – review and editing (equal). **Vivian Piedrahita:** data curation (supporting), methodology (supporting), project administration (supporting), supervision (lead), writing – review and editing (supporting). **Santiago Bolivar:** writing – review and editing (supporting). **Karina Grillo:** writing – review and editing (supporting). **Adriana Linares:** writing – review and editing (supporting). **Carlos Pardo:** writing – review and editing (supporting). **Martha Piña:** writing – review and editing (supporting). **Amaranto Suarez:** writing – review and editing (supporting). **Carlos A. Portilla:** funding acquisition (supporting), writing – review and editing (supporting). **Jesus Ardila:** funding acquisition (supporting), writing – review and editing (supporting). **Viviana Lotero:** writing – review and editing (supporting). **Luz A. Urcuqui:** writing – review and editing (supporting). **Angela Trujillo:** writing – review and editing (supporting). **Patricia Montenegro:** writing – review and editing (supporting). **Luis E. Bravo:** conceptualization (equal), funding acquisition (equal), methodology (supporting), software (supporting), writing – review and editing (supporting). **Paula Aristizabal:** conceptualization (supporting), formal analysis (supporting), funding acquisition (equal), methodology (supporting), project administration (supporting), supervision (supporting), writing – original draft (equal), writing – review and editing (equal).

## Ethics Statement

Approvals by Institutional Review Boards at the Universidad del Valle and participating sites were obtained.

## Conflicts of Interest

The authors declare no conflicts of interest.

## Data Set

Stata file: COVID childhood cancer dataset August 2022.dta/August 2022/Author: Oscar Ramirez/Fundación POHEMA repository.

## Supporting information


Table S1:

## Data Availability

The de‐identified data for this manuscript will be available on request from the corresponding author due to privacy/ethical restrictions.

## Appendix 1

**VIGICANCER working group collaborators at participating cities**:

**Table:**

*First name and middle initial(s)	*Last name	Academic degrees	Institution	Location (city, state/province, country)	Role or contribution
Gisella	Barros	MD; Pediatric Oncologist	Fundación Hospital Pediátrico la Misericordia	Bogotá, D.C., Colombia	Data acquisition coordination
Edgar	Cabrera‐Bernal	MD; Pediatric Oncologist	Fundación Hospital Pediátrico la Misericordia	Bogotá, D.C., Colombia	Data acquisition coordination
Angélica	Castillo	MD; Pediatric Oncologist	Clínica Infantil de Colsubsidio	Bogotá, D.C., Colombia	Data acquisition coordination
Mauricio	Chaparro	MD; Pediatric Oncologist	Fundación Hospital Pediátrico la Misericordia	Bogotá, D.C., Colombia	Data acquisition coordination
Marcela	Estupiñán	MD; Pediatric Oncologist	Fundación Hospital Pediátrico la Misericordia	Bogotá, D.C., Colombia	Data acquisition coordination
Jessica	Flechas	MD; Pediatric Oncologist	Fundación Hospital Pediátrico la Misericordia	Bogotá, D.C., Colombia	Data acquisition coordination
Jimmy	Lagos	MD; Pediatric Oncologist	Hospital Universitario San Ignacio	Bogotá, D.C., Colombia	Data acquisition coordination
Jorge	Hernández	MD; Pediatric Oncologist	Instituto Nacional de Cancerología	Bogotá, D.C., Colombia	Data acquisition coordination
John	Lopera	MD; Pediatric Oncologist	Instituto Nacional de Cancerología	Bogotá, D.C., Colombia	Data acquisition coordination
Ingrid	Aristizabal	MD; Pediatric Oncologist	Instituto Nacional de Cancerología	Bogotá, D.C., Colombia	Data acquisition coordination
Agustín	Contreras	MD; Pediatric Oncologist	Hospital de la Policía	Bogotá, D.C., Colombia	Data acquisition coordination
Iliana C.	De los Reyes	MD; Pediatric Oncologist	Hospital Militar Central	Bogotá, D.C., Colombia	Data acquisition coordination
Natalia	González	MD; Pediatric Oncologist	Hospital Militar Central	Bogotá, D.C., Colombia	Data acquisition coordination
Paula C.	Guzmán	MD; Pediatric Oncologist	Hospital Militar Central	Bogotá, D.C., Colombia	Data acquisition coordination
Alejandra	Calderón	MD; Pediatric Oncologist	Fundación Santa Fé de Bogotá	Bogotá, D.C., Colombia	Data acquisition coordination
Oscar	González	MD; Pediatric Oncologist	Fundación Santa Fé de Bogotá	Bogotá, D.C., Colombia	Data acquisition coordination
Mauricio	Mesa	MD; Pediatric Oncologist	Fundación Santa Fé de Bogotá	Bogotá, D.C., Colombia	Data acquisition coordination
Carolina	Casas	MD; Pediatric Oncologist	Hospital Universitario San Ignacio	Bogotá, D.C., Colombia	Data acquisition coordination
Ana M.	Infante	MD; Pediatric Oncologist	Hospital Universitario San Ignacio	Bogotá, D.C., Colombia	Data acquisition coordination
Leila	Martínez	MD; Pediatric Oncologist	Clínica Infantil de Colsubsidio	Bogotá, D.C., Colombia	Data acquisition coordination
Angelica	Castillo	MD; Pediatric Oncologist	Clínica Infantil de Colsubsidio	Bogotá, D.C., Colombia	Data acquisition coordination
Giovanny	Rincón	MD; Pediatric Oncologist	Hospital Infantil Universitario de San José	Bogotá, D.C., Colombia	Data acquisition coordination
Diego I.	Estupinan‐Perico	MD; Pediatric Oncologist	Clínica Materno Infantil San Luis	Bucaramanga, Colombia	Data acquisition coordination
María P.	Obregón	MD; Pediatric Oncologist	Clínica Materno Infantil San Luis	Bucaramanga, Colombia	Data acquisition coordination
David J.	Garay	MD; Pediatric Oncologist	Fundación Oftalmológica de Santander	Bucaramanga, Colombia	Data acquisition coordination
Julio A.	Ropero	MD; Pediatric Oncologist	Fundación Oftalmológica de Santander	Bucaramanga, Colombia	Data acquisition coordination
María A.	Pérez Sotelo	MD; Pediatric Oncologist	Fundación Cardiovascular de Colombia	Bucaramanga, Colombia	Data acquisition coordination
Liliana	Barragán	MD; Pediatric Oncologist	Clínica de Occidente	Cali, Colombia	Data acquisition coordination
Remberto	Osuna	MD; Pediatric Oncologist	Clínica de Occidente	Cali, Colombia	Data acquisition coordination
María X.	Castro	MD; Pediatric Oncologist	Hospital Universitario Fundación Valle del Lili	Cali, Colombia	Data acquisition coordination
Pamela A.	Rodríguez	MD; Pediatric Oncologist	Hospital Universitario Fundación Valle del Lili	Cali, Colombia	Data acquisition coordination
Diego	Medina	MD; Pediatric Oncologist	Hospital Universitario Fundación Valle del Lili	Cali, Colombia	Data acquisition coordination
Alexis	Franco	MD; Pediatric Oncologist	Hospital Universitario Fundación Valle del Lili	Cali, Colombia	Data acquisition coordination
Luis H.	Romero	MD; Pediatric Oncologist	Hospital Universitario del Valle Evaristo García	Cali, Colombia	Data acquisition coordination
Ruth	Castro	MD; Pediatric Oncologist	Clínica Imbanaco de Cali grupo Quirón Salud	Cali, Colombia	Data acquisition coordination
Carlos E.	Narváez	MD; Pediatric Oncologist	Clínica Imbanaco de Cali grupo Quirón Salud	Cali, Colombia	Data acquisition coordination
Diana S.	Rendón	MD; Pediatric Oncologist	Clínica Imbanaco de Cali grupo Quirón Salud	Cali, Colombia	Data acquisition coordination
Ángel	Castro	MD; Pediatric Oncologist	Clínica Blas de Lezo	Cartagena, Colombia	Data acquisition coordination
Ayslin	González	MD; Pediatric Oncologist	Fundación Hospital Infantil Napoleón Franco Pareja—La Casa del NIño	Cartagena, Colombia	Data acquisition coordination
Heidy	Marsiglia	MD; Pediatric Oncologist	Fundación Hospital Infantil Napoleón Franco Pareja—La Casa del NIño	Cartagena, Colombia	Data acquisition coordination
Soraya	Paternina	MD; Pediatric Oncologist	Fundación Hospital Infantil Napoleón Franco Pareja—La Casa del NIño	Cartagena, Colombia	Data acquisition coordination
Francy H.	Ortiz	MD; Pediatric Oncologist	Hospital Federico Lleras Acosta	Ibagué, Colombia	Data acquisition coordination
Cindy	Martínez	MD; Pediatric Oncologist	Clínica Infantil de Colsubsidio	Bogotá, D.C., Colombia	Data acquisition coordination
Bibiana	Villa	MD; Pediatric Oncologist	Oncólogos de Occidente	Manizales, Colombia	Data acquisition coordination
Javier E	Fox	MD; Pediatric Oncologist	Hospital San Vicente Fundación de Medellín	Medellín, Colombia	Data acquisition coordination
María R.	Pérez	MD; Pediatric Oncologist	Hospital San Vicente Fundación de Medellín	Medellín, Colombia	Data acquisition coordination
Carlos E.	Restrepo	MD; Pediatric Oncologist	Hospital San Vicente Fundación de Medellín	Medellín, Colombia	Data acquisition coordination
Lina M.	Quiroz	MD; Pediatric Oncologist	Hospital Pablo Tobón Uribe	Medellín, Colombia	Data acquisition coordination
Natalia	Valencia	MD; Pediatric Oncologist	Hospital Pablo Tobón Uribe	Medellín, Colombia	Data acquisition coordination
Alexandra	Restrepo	MD; Pediatric Oncologist	Hospital Pablo Tobón Uribe	Medellín, Colombia	Data acquisition coordination
Federico	Arroyave	MD; Pediatric Oncologist	Hospital Pablo Tobón Uribe	Medellín, Colombia	Data acquisition coordination
Hernán D.	Vásquez	MD; Pediatric Oncologist	Hospital General de Medellín	Medellín, Colombia	Data acquisition coordination
Gloria E.	Suarez	MD; Pediatric Oncologist	Instituto de Cancerología Las Américas, Clínica las Américas Auna	Medellín, Colombia	Data acquisition coordination
Diana L.	Valencia	MD; Pediatric Oncologist	Clínica IMAT Oncomedica Auna	Montería, Colombia	Data acquisition coordination
Fabio J.	Molina	MD; Pediatric Oncologist	Clínica IMAT Oncomedica Auna	Montería, Colombia	Data acquisition coordination
Soraya	Montaño	MD; Pediatric Oncologist	Clínica IMAT Oncomedica Auna	Montería, Colombia	Data acquisition coordination
Aide	Peña	MD; Pediatric Oncologist	Clínica IMAT Oncomedica Auna	Montería, Colombia	Data acquisition coordination
José A.	Cabezas	MD; Pediatric Oncologist	Hospital Universitario Hernando Moncaleano Perdomo	Neiva, Colombia	Data acquisition coordination
Nelson	Ramírez	MD; Pediatric Oncologist	Hospital Universitario Hernando Moncaleano Perdomo	Neiva, Colombia	Data acquisition coordination
Maria del Rosario	Álvarez	MD; Pediatric Oncologist	Hospital Infantil Los Ángeles	Pasto, Colombia	Data acquisition coordination
