# Supplementary material for: Increased Early‐Mortality in Children With Solid Tumors During the COVID‐19 Pandemic in a Middle‐Income Country
Source: Cancer Med. 2024 Dec 23;13(24):e70483. doi: 10.1002/cam4.70483 (PMC11803909; doi:10.1002/cam4.70483)
Supplement: Supplementary file 1 — Table S1: [file CAM4-13-e70483-s001.docx]

**Supplemental Content**

**Supplementary Table 1.** Distribution of Participants by ICCC Tumoral Group and Cohort

**Supplementary Table 2.** Adjusted OR for Metastatic Disease by Cohort and Sociodemographic Characteristics

| **Tumoral group** | | **Exposed** | | | **Historical** | | **Total** | |
| --- | --- | --- | --- | --- | --- | --- | --- | --- |
|  |  | **cohort** | | **cohort** | | |  |  |
|  |  | **n** | **(%)^b^** | **n** | | **(%)** | **N** | **(%)** |
| I. | Leukemias | 738 | (45) | 1132 | | (45) | 1870 | (45) |
| II. | Lymphomas | 185 | (11) | 290 | | (12) | 475 | (12) |
| III. | Primary CNS tumors | 245 | (15) | 376 | | (15) | 621 | (15) |
| IV. | Neuroblastomas | 40 | (3) | 79 | | (3) | 119 | (3) |
| V. | Retinoblastomas | 57 | (4) | 77 | | (3) | 134 | (3) |
| VI. | Renal tumors | 78 | (5) | 117 | | (5) | 195 | (5) |
| VII. | Hepatic tumors | 27 | (2) | 45 | | (2) | 72 | (2) |
| VIII. | Malignant bone tumors | 106 | (7) | 148 | | (6) | 254 | (6) |
| IX. | Soft tissue sarcomas | 56 | (3) | 101 | | (4) | 157 | (4) |
| X. | Germ cell tumors | 61 | (4) | 81 | | (3) | 142 | (3) |
| XI. | Other epithelial and melanomas | 30 | (2) | 47 | | (2) | 77 | (2) |
| XII. | Other and unspecified | 4 | (0) | 4 | | (0) | 8 | (0) |
|  | Total^c^ | 1627 | (39) | 2497 | | (61) | 4124 | (100) |

**Supplementary Table 1.** Distribution of Participants by ICCC-3 Tumoral Group and Cohort^a^

Abbreviations: ICCC-3, international classification of childhood cancer; CNS, central nervous system.

^a^Exposed cohort, 2020-2021; historical cohort, 2017-2019.

^b^We use the cohort's total number (column totals) as the denominator to calculate the percentage.

^c^Of note, the sum of some of the totals may not be exactly 100% due to rounding.

**Supplementary Table 2.** Adjusted OR for Metastatic Disease by Cohort^a^ and Sociodemographic Characteristics

| **Characteristics^b^** | | **Exposed cohort** | | |  | **Historical cohort** | | | **N** | **aOR^d^ (95%CI)** |
| --- | --- | --- | --- | --- | --- | --- | --- | --- | --- | --- |
|  |  | **n** | **m** | **(%)^c^** |  | **n** | **m** | **(%)** |  |  |
| Age group (years) | |  | |  |  |  | |  |  |  |
|  | <10 | 215 | 75 | 35 |  | 421 | 119 | 28 | 636 | 1.5 (1.0-2.1) |
|  | 10-14 | 160 | 65 | 41 |  | 188 | 72 | 38 | 348 | 1.3 (0.8-2.0) |
| Sex | |  | |  |  |  | |  |  |  |
|  | Male | 193 | 75 | 39 |  | 314 | 104 | 33 | 507 | 1.4 (0.9-2.1) |
|  | Female | 182 | 65 | 36 |  | 295 | 87 | 29 | 477 | 1.5 (1.0-2.2) |
| Afro-descendant ethnicity (N=983) | |  |  |  |  |  | |  |  |  |
|  | Yes | 24 | 13 | 54 |  | 47 | 12 | 26 | 71 | 3.4 (1.0-12.1) |
|  | No | 351 | 127 | 36 |  | 561 | 179 | 32 | 912 | 1.3 (1.0-1.7) |
| Residing in a city with a POU | |  |  |  |  |  | |  |  |  |
|  | Yes | 149 | 46 | 31 |  | 247 | 76 | 31 | 396 | 1.0 (0.7-1.7) |
|  | No | 226 | 94 | 42 |  | 362 | 115 | 32 | 588 | 1.7 (1.2-2.4) |
| Cities (new cancer cases/year) | |  |  |  |  |  |  |  |  |  |
|  | >100 | 309 | 118 | 38 |  | 485 | 155 | 32 | 794 | 1.4 (1.0-1.9) |
|  | <100 | 66 | 22 | 33 |  | 124 | 36 | 29 | 190 | 1.4 (0.7-2.7) |
| Health insurance affiliation^d^ | |  |  |  |  |  |  |  |  |  |
|  | Public | 174 | 69 | 40 |  | 248 | 86 | 35 | 422 | 1.2 (0.8-1.8) |
|  | Semi-private | 176 | 65 | 37 |  | 292 | 77 | 26 | 468 | 1.7 (1.1-2.6) |

Abbreviation: aOR, adjusted odds ratio estimated by conditional logistic regression, adjusted for age, sex, Afro-descendant ethnicity, and place of residence, grouped by city. The aOR is estimated by comparing the exposed vs. the historical cohort (reference) for each stratum of the independent variables included in the table; n, total number of participants with information about metastatic status; m, number of participants with metastatic disease at diagnosis; N, number of observations in each logistic model.

^a^Exposed cohort, March 25, 2020, to Dec 31, 2021; historical cohort, Jan 1, 2017, to March 24, 2020.

^b^984 is the total number of participants unless otherwise indicated. 15% of patients (n=174) were not included in the analysis due to data missing regarding metastatic status.

^c^We use the total number of participants with information about metastatic status (n) for each cohort in each independent variable category, as the denominator to calculate the percentage for metastatic disease (m).

^d^Private/special plans and uninsured categories are not shown due to their low number of participants.
